# Instrumental variable estimation in semi‐parametric additive hazards models

**DOI:** 10.1111/biom.12952

**Published:** 2018-08-02

**Authors:** Matthias Brueckner, Andrew Titman, Thomas Jaki

**Affiliations:** ^1^ Department of Mathematics and Statistics Lancaster University Lancaster LA1 4YF U.K.

**Keywords:** Additive hazard, Confounding, Instrumental variable, Survival analysis

## Abstract

Instrumental variable methods allow unbiased estimation in the presence of unmeasured confounders when an appropriate instrumental variable is available. Two‐stage least‐squares and residual inclusion methods have recently been adapted to additive hazard models for censored survival data. The semi‐parametric additive hazard model which can include time‐independent and time‐dependent covariate effects is particularly suited for the two‐stage residual inclusion method, since it allows direct estimation of time‐independent covariate effects without restricting the effect of the residual on the hazard. In this article, we prove asymptotic normality of two‐stage residual inclusion estimators of regression coefficients in a semi‐parametric additive hazard model with time‐independent and time‐dependent covariate effects. We consider the cases of continuous and binary exposure. Estimation of the conditional survival function given observed covariates is discussed and a resampling scheme is proposed to obtain simultaneous confidence bands. The new methods are compared to existing ones in a simulation study and are applied to a real data set. The proposed methods perform favorably especially in cases with exposure‐dependent censoring.

## 1 Introduction

Instrumental variables (IV) can be used in regression modeling to avoid bias from unmeasured confounding or dependent measurement error in covariates by providing a source of exogenous variation (Angrist et al., 1996). These methods are also popular in epidemiology in the analysis of observational studies. In randomized clinical trials with survival endpoints unmeasured confounding may occur as a result of non‐compliance, for example, when patients switch to salvage treatment after a progression of the disease. Applying naive analysis methods in such circumstances may result in severe bias (Zeng et al., 2012).

Two‐stage IV methods for duration data in econometrics have been proposed by Bijwaard and Ridder (2005). Estimation of survival probabilities under treatment non‐compliance using IV methods was considered by Nie et al. (2011). Baker (1998) estimates life years saved using IV methods in the context of all‐or‐none compliance. Two‐stage IV methods for parametric Bayesian models have been developed by Li and Lu (2015), and non‐parametric binary IV methods for competing risks data by Richardson et al. (2017). The additive hazard model (Aalen, 1989) is particularly amenable to IV methods, since it resembles the linear regression model, while the popular Cox proportional hazards model is inappropriate for IV methods as shown by Tchetgen Tchetgen et al. (2015).

For additive hazard survival models with censored data several two‐stage methods employing IVs have been developed. In the two‐stage least squares (2SLS) method, the first stage consists of a linear model for the confounded exposure given the IV and other observed covariates. In the second stage, an additive hazard model is fitted with the observed exposure being replaced by the predicted exposure from the first stage regression. Alternatively, the two‐stage residual inclusion (2SRI) method (Terza et al., 2008) keeps the observed exposure in the model, but includes the estimated first stage residual as additional covariate in the model.

For the 2SRI method, the first stage does not need to be a linear model, but additional assumptions about the unobserved confounding are required (Tchetgen Tchetgen et al., 2015). Essentially, in the case of continuous exposure, it is required that the unobserved confounding is a linear function of the first stage residual plus an independent error term. In the case of binary exposure we must be able to write the unobserved confounder as the sum of the conditional expectation of the unobserved confounder given exposure, instrument and observed covariates and an independent error term. These assumptions will be detailed in Section 2.

A 2SLS method for a continuous instrument for the semi‐parametric additive hazard model of Lin and Ying (1994), where all covariate effects are assumed to be time‐independent, was developed by Li et al. (2015). A similar 2SLS method for continuous instruments was proposed by Tchetgen Tchetgen et al. (2015) for the non‐parametric additive hazard model of Aalen (1989), where all covariate effects are allowed to be time‐dependent. For the same model they also develop a 2SRI method for binary and continuous instruments. However, asymptotic results are only provided for the 2SLS method. Work on IV methods for the additive hazard model has focused on the case of only time‐independent covariate effects. The semi‐parametric additive hazards model of McKeague and Sasieni (1994), which allows time‐independent and time‐dependent effects has received less attention. We argue that this model is more appropriate for the 2SRI method, since it does not require the effect of the residual included in the second stage model to be time‐independent. At the same time the exposure effect can still be modeled as time‐independent, which may be more useful to summarize treatment effects in a randomized trial.

While the 2SRI method requires more stringent assumptions about the influence of the unobserved confounder on the hazard, the assumptions about the censoring can be relaxed. It is sufficient that the censoring is independent of the survival time conditional on the exposure and observed covariates, since the exposure is still part of the model (Chan, 2016). While the 2SLS method with a linear first stage can be used in the case of a binary exposure, a non‐linear first stage model, such as a logistic regression model, might be more appropriate.

A different and very general approach is taken by Martinussen et al. (2017), who develop an IV method for a class of structural cumulative survival models. Their approach does not require any modeling of the relationship between the exposure and the instrument. However, it requires a parametric model for conditional expectation of the instrument given the observed confounders and the survival function cannot be readily estimated from this model. In recent work Choi et al. (2017) proposed a two‐stage procedure for general structural equation models, that can also be applied to censored survival data.

In Section 2, we extend the 2SRI methods for continuous and binary exposure to the semi‐parametric additive hazards model of McKeague and Sasieni (1994), which allows for time‐dependent and time‐independent covariate effects. Hence, the residual can be included in the model without restrictions (other than linearity), while the effect of the other covariates can be modeled as time‐independent. Asymptotic results are derived for the 2SRI approach with binary and continuous exposure and instrument. In Section 2.3, an iid decomposition of an estimator of the conditional survival function given the exposure, the instrument and all observed confounders is proved. Based on this result a resampling scheme for obtaining simultaneous confidence bands is proposed. In our simulation study in Section 3, we find the 2SRI method to be superior to the 2SLS in the binary case and/or exposure‐dependent censoring for the survival times. In Section 4, the methods are applied to a dataset from the Illinois unemployment bonus experiment (Woodbury and Spiegelman, 1987), where participants receiving unemployment benefits were offered a cash bonus on re‐employment.

## 2 Two‐Stage Instrumental Variable Methods

Let *T* be a continuous survival time, *C* the censoring time, and Y=min{T,C} the observed right‐censored survival time. We assume that the follow‐up period is a fixed finite interval [0,τ] and that the hazard of *T* follows an additive hazard model

(1) $$\begin{array}{ccc} h(t|R,L,U) & = & \alpha_{0}(t)+\beta_{R}R+\beta_{L}^{\prime }L_{Z}+\alpha_{L}(t)L_{X}\\ & & +\alpha_{U}(U,t)\;(0\leq t\leq \tau ), \end{array}$$

where $\alpha_{0}$ is the baseline hazard, *R* is the observed exposure/treatment indicator with a time‐independent effect, $L_{Z}$ is a *p*‐vector of observed covariates with time‐independent effects, $L_{X}$ is a *q*‐vector of observed covariates with time‐dependent effects, and $\alpha_{U}(U,t)$ is a term depending on a vector of unobserved confounders *U*. All covariates in the model are baseline covariates which cannot change over time. We call this model the “McKeague–Sasieni model” (McKeague and Sasieni, 1994). The additive hazard model of Lin and Ying (1994) where all covariate effects are time‐independent will be called the “Lin–Ying model”. The original additive hazard model of Aalen (1989) where all covariate effects are unrestricted will be called the “Aalen model”. Both the Lin–Ying and the Aalen model can be viewed as special cases of the McKeague–Sasieni model.

Our main focus is on estimating the causal effect of the exposure on the hazard $\beta_{R}$. In general IV, methods can only identify the local average treatment effect (LATE) as shown in Angrist et al. (1996), that is, the average treatment effect of those whose exposure changes when the value of the IV changes. IV methods cannot say anything about subjects whose exposure is always the same regardless of the value of the IV (so‐called “always‐takers” and “never‐takers” in the context of binary treatment assignment and instrument). However, implicit in Model (1) is the assumption that the treatment effect $\beta_{R}$ is the same for all individuals for a given value of the covariates. This means that the LATE is equal to $\beta_{R}$ for all subjects and can therefore be interpreted as the average treatment effect (ATE) for the entire population. Hence, the IV estimate in this model is a consistent estimate of the population ATE.

Alternatively, one could start with the Aalen model and then use

$${\hat{\beta }}_{R}=\frac{1}{\tau }\int_{0}^{\tau }{\hat{B}}_{R}(t)\mathrm{dt}$$

as an estimate of $\beta_{R}$, where τ is a fixed time horizon and ${\hat{B}}_{R}(t)$ is a consistent estimate of the cumulative effect $\int_{0}^{t}\beta_{R}(s)\mathrm{ds}$ obtained by 2SLS or 2SRI in the Aalen model (Tchetgen Tchetgen et al., 2015). Outside of the two‐stage setting this approach was also taken by Martinussen et al. (2017). However, this estimate would have a larger standard error than the semi‐parametric estimate and τ may not be data dependent.

Let $L={\left(L_{Z}^{\prime },L_{X}^{\prime }\right)}^{\prime }$. Formally we assume the existence of an instrumental variable *G*, such that following assumptions hold:

A1 *G* is associated with *R* conditional on *L*.
A2 *G* is independent of *T* conditional on *L*, *R*, and *U*.

Assumption (A1) implies that there is a non‐zero average causal effect of the instrument *G* on the exposure *R* and Assumption (A2) is the exclusion restriction of Angrist et al. (1996). We also assume that *L* and *G* are exogenous, that is,

A3 *U* is independent of *L* and *G*.

The 2SLS methods of Li et al. (2015) and Tchetgen Tchetgen et al. (2015) first predict the exposure from a linear regression model given the instrument and any observed covariates. Then an additive hazard model is fitted with the observed exposure replaced by the predicted exposure. In the 2SRI method of Tchetgen Tchetgen et al. (2015), the observed exposure is kept and instead the residual of the first stage regression is included as an additional regressor in the second stage model. For uncensored observations and linear first and second stage models both methods would coincide. However, in the case of a binary exposure a non‐linear first stage model, such as a logistic regression model, might be more appropriate.

When considering regression methods for censored survival data it is usually necessary to assume independence of censoring and survival times conditional on all covariates included in the model. The 2SLS method requires censoring *C* and survival time *T* to be independent conditional on the observed covariates *L*. The 2SLS method can suffer from bias when censoring and survival are dependent on the exposure *R*. The bias of the 2SLS method induced by exposure dependent censoring is explored in Scenario VI of Li et al. (2015) and in our own simulations in Section 3. Since the exposure *R* is still included in the second stage model, it is sufficient to require conditional independence of censoring and survival times given the observed covariates and the exposure (Chan, 2016):

A4 *C* is independent of *T* conditional on *R* and *L*.

**Figure 1:**
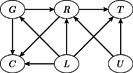
Visualization of IV assumptions (A1)–(A4) with instrument *G*, exposure *R*, survival time *T*, observed confounders *L*, unobserved confounders *U*, and censoring time *C*

The relationships encoded in Assumptions (A1)–(A4) can be represented by a directed acyclic graph (DAG) as shown in Figure 1. The arrows represent dependencies between random variables. There is an arrow from *G* to *R* (Assumption (A1)), but no arrow from *G* to *T* (Assumption (A2)) and no arrows from *U* to *L* and *G* (Assumption (A3)). The censoring *C* is allowed to depend on the instrument *G* for 2SRI, since removing the nodes *R* and *L* from the DAG separates *T* and *C* even when *C* depends on *G*. It is however important to note that *C* must be independent from the unobserved confounder *U* given *R* and *L*, that is, no arrow from *U* to *C*.

### 2.1 Binary Case

In the case of a binary exposure *R* we use a logistic regression model in the first stage

(2) $$\text{log}\left(\frac{p}{1-p}\right)=\gamma_{0}+\gamma_{G}G+\gamma_{L}^{\prime }L$$

where $\gamma =(\gamma_{0},\gamma_{G},\gamma_{L}^{\prime })$ and p=P(R=1|G,L). Denote the maximum likelihood estimator of γ by $\hat{\gamma }$. The predicted probability for a patient with instrument *G* and covariates *L* from this model is

$$\hat{p}=\frac{1}{1+\text{exp}\{-{\hat{\gamma }}^{\prime }{\left(G,L^{\prime }\right)}^{\prime }\}}.$$

The 2SRI method requires an additional linearity assumption about the unobserved heterogeneity (Tchetgen Tchetgen et al., 2015):

A5 $\alpha_{U}(U,t)=E\left\{\alpha_{U}(U,t)|R,G,L\right\}+\varepsilon (t)$,

where ε(t) is an error independent of *R*, *G* and *L*. This assumption holds, for example, when *U* has a normal distribution where only the mean depends on *R*, *G* and *L*.

Under assumptions (A1)–(A5) a reparametrization of the original model can be obtained from Result 3 of Tchetgen Tchetgen et al. (2015):

(3) $$\begin{array}{ccc} h(t|R,G,L) & = & {\tilde{\alpha }}_{0}(t)+\beta_{R}R+\beta_{L}^{\prime }L_{Z}+\alpha_{L}{\left(t\right)}^{\prime }L_{X}\\ & & +\left\{\rho_{0}(t)+\rho_{1}(t)G\right\}\Delta , \end{array}$$

where Δ≡Δ(R,G,L)=R−P(R=1|G,L), $\rho_{0}(t)=E\{\alpha_{U}(U,t)|R=1,G=0,L\}-E\{\alpha_{U}(U,t)|R=0,G=0,L\}$, and $\rho_{1}(t)\;=E\{\alpha_{U}(U,t)|R\;=\;1,G\;=\;1,L\}-E\{\alpha_{U}(U,t)|R=0,G=0,L\}-\rho_{0}(t)$. Since the true residual Δ is unknown it is estimated by $\hat{\Delta }=R-\hat{p}$.

We emphasize, that the conditional independence assumption (A4) is sufficient in the binary exposure case as well, that is, censoring is allowed to be dependent on the binary exposure.

An interesting special case is when the exposure is confounded only for the group with G=1, which implies that *U* is independent of *R* given G=0 and *L*. This is the case in our data example (Section 4) with full compliance in the control group. In this case $\rho_{0}\equiv 0$ and the conditional hazard becomes

(4) $$h(t|R,G,L)={\tilde{\alpha }}_{0}(t)+\beta_{R}R+\beta_{L}^{\prime }L_{Z}+\alpha_{L}{\left(t\right)}^{\prime }L_{X}+\rho_{1}(t)G\Delta .$$

If instead *U* is independent of *R* given G=1 and *L*, then $\rho_{1}=-\rho_{0}$ and $\rho_{1}(t)G\Delta$ is replaced by $\rho_{1}(t)(1-G)\Delta$ in equation (4). For example, such a situation occurred in the panitumumab colorectal cancer trial (Amado et al., 2008), where patients randomized to the standard of care group had the possibility of switching to the experimental treatment on disease progression. Fitting the model which only includes the residual‐instrument interaction but not the main effect of the residual may avoid numerical stability issues as in our data example (Section 4).

We are interested in estimating the vector of regression coefficients $\beta ={\left(\beta_{R},\beta_{L}^{\prime }\right)}^{\prime }$ and the vector of cumulative covariate effects

$$A(t)=\int_{0}^{t}{\left\{{\tilde{\alpha }}_{0}(s),\alpha_{L}{\left(s\right)}^{\prime },\rho_{0}(s),\rho_{1}(s)\right\}}^{\prime }\mathrm{ds}.$$

Let Z=Z(t) be the n×(p+1) matrix with *i*‐th row given by $Y_{i}(t)(R_{i},L_{\mathrm{Zi}}^{\prime })$, where $Y_{i}(t)=I(Y_{i}\geq t)$ is the at‐risk indicator at time *t* of the *i*‐th subject. The n×(q+3) design matrix X=X(t) for the time‐dependent coefficient functions including the baseline hazard function is defined like *Z* with *i*‐th row equal to $Y_{i}(t)(1,L_{\mathrm{Xi}}^{\prime },\Delta_{i},\Delta_{i}G_{i})$. Furthermore, we obtain the matrix $\hat{X}=\hat{X}(t)$ by replacing in *X* the unknown residuals Δ with the estimated residuals $\hat{\Delta }$. We can then define the estimators of β and *A* like those given by McKeague and Sasieni (1994), but using $\hat{X}$ instead of *X*,

(5) $$\hat{\beta }={\left(\int_{0}^{\tau }Z^{\prime }\hat{H}\mathrm{Zdt}\right)}^{-1}\int_{0}^{\tau }Z^{\prime }\hat{H}\mathrm{dN},$$

and

(6) $$\hat{A}(t)=\int_{0}^{t}{\left({\hat{X}}^{\prime }\hat{X}\right)}^{-1}({\hat{X}}^{\prime }\mathrm{dN}-{\hat{X}}^{\prime }Z\hat{\beta }\mathrm{ds}),$$

where $\hat{H}=I-\hat{X}{\left({\hat{X}}^{\prime }\hat{X}\right)}^{-1}{\hat{X}}^{\prime }$, *I* is the (q+3)×(q+3) identity matrix and $N(t)=\{N_{1}(t),\ldots ,N_{n}(t){\;\}}^{\prime }=\{I(Y_{1}\leq t)\delta_{1},\ldots ,I(Y_{n}\leq t)\delta_{n}{\;\}}^{\prime }$ is the vector of counting processes.

The additional variation in the second stage introduced by $\hat{X}$ must be taken into account when calculating standard errors for the regression coefficients. The correct standard errors are given by Theorem 2.1 below. Its proof and the required regularity assumptions (B1)–(B6) are given in the Appendix.

Theorem 1

1. Under the IV assumptions (A1)–(A5) and the regularity assumptions (B1)–(B3) we have
  (7) $$\sqrt{n}(\hat{\beta }-\beta )=n^{-1/2}\sum_{i=1}^{n}\varepsilon_{i}^{\left(\beta \right)}+o_{p}(1),$$
  where $\varepsilon_{i}^{\left(\beta \right)}$ are iid vectors defined in equation (A.5) in the Appendix. This implies that $\hat{\beta }=\beta +o_{p}(1)$ and $\sqrt{n}(\hat{\beta }-\beta )$ is asymptotically normal with mean zero and covariance matrix $\Sigma_{\beta }=E(\varepsilon_{i}^{(\beta )⊗2})$, where $a^{⊗2}={\mathrm{aa}}^{\prime }$ for a vector *a*.
2. Under assumptions (A1)–(A5) and (B1)–(B6) we have
  (8) $$\sqrt{n}(\hat{A}-A)=n^{-1/2}\sum_{i=1}^{n}\varepsilon_{i}^{\left(A\right)}+o_{p}(1),$$
  where $\varepsilon_{i}^{\left(A\right)}$ are iid functions defined in equation (A.7) in the Appendix. This implies that $\underset{t}{\text{sup}}∥\hat{A}(t)-A(t)∥=o_{p}(1)$ and $\sqrt{n}(\hat{A}-A)$ converges weakly to a vector of mean‐zero Gaussian processes with covariance function $\Sigma_{A}(s,t)=E\{\varepsilon_{i}^{\left(A\right)}(s)\varepsilon_{i}^{\left(A\right)}{\left(t\right)}^{\prime }\}$.

Theorem 2.1 can also be applied in the less restrictive Aalen model

(9) $$\begin{array}{ccc} h(t|R,G,L) & = & {\tilde{\alpha }}_{0}(t)+\{\alpha_{R}(t),\alpha_{L}{\left(t\right)}^{\prime }\}{\left(R,L_{X}^{\prime }\right)}^{\prime }\\ & & +\rho_{0}(t)\Delta +\rho_{1}(t)\Delta G, \end{array}$$

with only time‐dependent covariate effects by setting Z=0, which implies Ψ(t)=0 for all *t*.

### 2.2 Continuous Case

For a continuous exposure we assume a linear model as the first stage model, that is,

$$R=\gamma_{0}+\gamma_{G}G+\gamma_{L}^{\prime }L+\Delta .$$

Assumption (A5) needs to be modified to

A5c $\alpha_{U}(U,t)=\rho_{0}(t)\Delta +\varepsilon (t)$,

where ε(t) is an error term independent of Δ (Tchetgen Tchetgen et al., 2015). According to Result 2 of Tchetgen Tchetgen et al. (2015) we have

(10) $$h(t|R,G,L)={\tilde{\alpha }}_{0}(t)+\beta_{R}R+\beta_{L}^{\prime }L_{Z}+\alpha_{L}{\left(t\right)}^{\prime }L_{X}+\rho_{0}(t)\Delta .$$

When fitting this model the true unknown residual Δ is again replaced with the residual from the first stage regression $\hat{\Delta }=R-\hat{\gamma }{\left(1,G,L^{\prime }\right)}^{\prime }$. The result for the asymptotic distribution of Theorem 2.1 still holds, when we replace $u_{1i}(t)$ and $u_{2i}(t)$ with ${\tilde{u}}_{1i}(t)=Y_{i}(t)$ and ${\tilde{u}}_{2i}(t)\equiv 0$, respectively, in Assumption (B2) and (B6). As in the binary case, this holds for the special case of only time‐dependent effects (equation (9)) as well.

### 2.3 Estimation of the Conditional Survival Function

In the 2SLS approach, it is possible to estimate the survival function of *T* given *R* and *L* only, as shown by Li et al. (2015), whereas in the 2SRI approach this can only be achieved by further modeling of the conditional distribution of *G* given *R* and *L* and then taking the expectation of S(t|R,G,L) with respect to that distribution. This is because we can only estimate the covariate effects in the model for the conditional hazard h(t|R,L,U) (equation (1)), but we cannot estimate the original baseline hazard $\alpha_{0}(t)$. Therefore, the survival function can only be estimated from the model for the conditional hazard h(t|R,G,L) (equations (3) and (10)), which explicitly depends on the first stage residual and therefore on the instrument *G*. Only in the case of binary instrument and exposure and no covariates is a simple non‐parametric estimator of S(t|R) available:

$$\hat{S}(t|R=r)=\frac{\sum_{i=1}^{n}\hat{S}(t|R=r,G=g)I(G_{i}=g,R_{i}=r)}{\sum_{i=1}^{n}I(R_{i}=r)}.$$

Let $\delta (\gamma )=r-(1,g,l_{Z}^{\prime },l_{X}^{\prime })\gamma$ and $\bar{p}(r,g,l)=p(r,g,l)\{1-p(r,g,l)\}$, where $p(r,g,l)=1/[1+\exp\{-(1,r,g,l^{\prime })\gamma \}]$. Then

$$S(t|r,g,l_{Z}^{\prime },l_{X}^{\prime })=\text{exp}\left\{-x(\gamma )A(t)-t(\beta_{R}r+\beta_{L}^{\prime }l_{Z})\right\},$$

where $x(\gamma )=\{1,l_{X}^{\prime },\delta (\gamma )\}$ in the continuous and $x(\gamma )=\{1,l_{X}^{\prime },\delta (\gamma ),\delta (\gamma )g\}$ in the binary case. Uniform consistency and asymptotic normality of the obvious estimator

(11) $$\hat{S}(t|r,g,l_{Z},l_{X})=\text{exp}\left\{-x(\hat{\gamma })\hat{A}(t)-t({\hat{\beta }}_{R}r+{\hat{\beta }}_{L}^{\prime }l_{Z})\right\},$$

follow from a Taylor expansion around {γ,β,A(t)} and the iid decompositions given in Theorem 2.3.

In principle an estimator of $S(t|r,l_{Z},l_{X})$ could be obtained by

$$\hat{S}(t|r,l_{Z},l_{X})=\frac{1}{n}\sum_{i=1}^{n}\hat{S}(t|r,G_{i},l_{Z},l_{X})\hat{f}(G_{i}|r,l_{Z},l_{X}),$$

where $\hat{f}(G_{i}|r,l_{Z},l_{X})$ is an estimator of the conditional probability density of *G* given R=r and $L=(l_{Z}^{\prime },l_{X}^{\prime })$, such as a kernel density estimator, which is feasible when the dimension of the covariate vector *L* is small. However, deriving the asymptotic properties of $\hat{S}(t|r,l_{Z},l_{X})$ is beyond the scope of this article.

Theorem 2 Let $W_{n}(t)=\sqrt{n}\{\hat{S}(t|r,g,l_{Z},l_{X})-S(t|r,g,l_{Z},l_{X})\}$. Under assumptions (A1)–(A5) and (B1)–(B6) we have

$$W_{n}(t)=n^{-1/2}\sum_{i=1}^{n}\varepsilon_{i}(t,r,g,l_{Z},l_{X})+o_{p}(1),$$

where

$$\begin{array}{ccc} \varepsilon_{i}(t,r,g,l_{Z},l_{X}) & = & -S(t|r,g,l_{Z},l_{X})\left\{t(r,l_{Z}^{\prime })\varepsilon_{i}^{\left(\beta \right)}+x(\gamma )\varepsilon_{i}^{\left(A\right)}(t)\right.\\ & & \left.-(1,g,l^{\prime })A_{q+2}(t)\varepsilon_{i}^{\left(\gamma \right)}\right\} \end{array}$$

in the continuous case and

$$\begin{array}{ccc} \varepsilon_{i}(t,r,g,l_{Z},l_{X}) & = & -S(t|r,g,l_{Z},l_{X})\left[t(r,l_{Z}^{\prime })\varepsilon_{i}^{\left(\beta \right)}+x(\gamma )\varepsilon_{i}^{\left(A\right)}(t)\right.\\ & & \left.-\bar{p}(r,g,l)(1,g,l^{\prime })\left\{A_{q+2}(t)+{\mathrm{gA}}_{q+3}(t)\right\}\varepsilon_{i}^{\left(\gamma \right)}\right] \end{array}$$

in the binary case, respectively, are iid random variables. The iid decomposition implies weak convergence of $W_{n}$ to a Gaussian process whose variance function can be consistently estimated by $t\mapsto n^{-1}\sum_{i}{\hat{\varepsilon }}_{i}{\left(t,r,l_{Z},l_{X}\right)}^{⊗2}$, where ${\hat{\varepsilon }}_{i}(t,r,g,l_{Z},l_{X})$ is obtained by replacing all unknown quantities in the definition of $\varepsilon_{i}(t,r,g,l_{Z},l_{X})$ with their consistent estimators.

Theorem 2.3 follows from a Taylor expansion of $\hat{S}(t|r,g,l_{Z},l_{X})$ around (γ,β,A(t)) and the iid decompositions of $\sqrt{n}(\hat{\gamma }-\gamma )$, $\sqrt{n}(\hat{\beta }-\beta )$ and $\sqrt{n}(\hat{A}-A)$ in Theorem 2.1.

Simultaneous confidence bands for $S(t|r,g,l_{Z},l_{X})$ can be obtained by $\hat{S}(t|r,g,l_{Z},l_{X})\pm n^{-1/2}q_{\alpha }$, where $q_{\alpha }$ is such that $P(\underset{t}{\text{sup}}|W_{n}(t)|\leq q_{\alpha })=1-\alpha$. The distribution of $W_{n}(t)$ can be approximated using a resampling approach based on the iid decomposition in Theorem 2.3. For independent standard normal random variables $Q_{1}^{m},\ldots ,Q_{n}^{m}$, given the observed data,the process

$${\hat{W}}_{m}(t|r,g,l_{Z},l_{X})=n^{-1/2}\sum_{i=1}^{n}{\hat{\varepsilon }}_{i}(t,r,g,l_{Z},l_{X})Q_{i}^{m},$$

has the same asymptotic distribution as $W_{n}(t)$ (Theorem 5.4.1 Martinussen and Scheike, 2006). Therefore the limiting distribution of $W_{n}(t)$ can be approximated by the empirical distribution of ${\hat{W}}_{1},\ldots ,{\hat{W}}_{M}$ for a large number *M*. The quantile $q_{\alpha }$ is then obtained as the empirical quantile of $\underset{t}{\text{sup}}|{\hat{W}}_{1}(t)|,\ldots ,\underset{t}{\text{sup}}|{\hat{W}}_{M}(t)|$.

## 3 Simulations

We compare the finite‐sample properties of the benchmark method (all confounders included in the model), the two‐stage residual inclusion (2SRI) method, two‐stage least squares method and naive method (confounders ignored) in several simulation scenarios with continuous and binary exposure.

### 3.1 Scenarios

1. This scenario corresponds to Case I of Li et al. (2015). The instrument *G*, unobserved confounder *U* and observed confounder *L* are all standard normal. The exposure *R* is continuous and is generated from the linear model $R=1+0.5G+L+U+N(0,0.2^{2})$, where L ∼N(0,1). The conditional hazard of the survival time is h(t|R,L,U)=9.5+0.5R+0.5L+1.5U. The censoring time is exponential with rate 2.5.
2. Same as Scenario 1, but with exposure‐dependent censoring, that is, censoring time is now exponential with rate $2.5+0.5R^{2}$
3. Same as Scenario 1, but linearity condition (A5c) for the confounder violated, that is, in the first stage R=1+0.5G+L+Δ, where $\Delta ∼N(0,0.2^{2})$ and $U=\Delta^{2}+N(0,1+\Delta^{2})$.
4. Slight modification of Scenario 3 from Martinussen et al. (2017) with continuous instrument $G∼N(2,1.5^{2})$ and unobserved confounder $U=1.5Z^{2}$, where $Z∼N(1,0.25^{2})$. The binary exposure is generated from the logistic regression model
  logit{P(R=1|G,U)}=−1+0.2G+U−E(U).
  The conditional hazard of the survival time is h(t|R,U)=0.05+0.4R+0.3U and censoring is uniform on [0,5].
5. This scenario corresponds to Case VII from Li et al. (2015). The instrument is binary with P(G=1)=0.5. The unobserved confounder *U* is standard normal. The exposure is set to 1 if 1.5G+1.5U+ε≥0 and to 0 otherwise, where ε is normal with mean 0 and standard deviation 0.2. This corresponds to a probit model. The survival time has hazard $h(t|R,U)=11+\beta_{R}(t)R+1.5U$ where $\beta_{R}(t)=2.5$ for all *t* and censoring is exponential with rate 2.5.
6. Same as Scenario 5, but with exposure‐dependent censoring, that is, *C* given *R* has an exponential distribution with rate 1/{0.1(1−R)+0.3R}.

Our results include as special cases the additive hazards model where all effects are modeled as time‐dependent. We consider a scenario with time‐dependent exposure effect on the hazard.

7 The same as Scenario 4, but now $\beta_{R}(t)=2.5I(t<0.1)-2.5I(0.1\leq t<0.2)$.

In the scenarios with binary exposure estimates were only calculated up to times where at least 15 (approx. 3–4 times the number of covariates) subjects were still at‐risk, in order to avoid numerical instability with singular matrices in the calculation of the estimates.

### 3.2 Results

In this section, we consider the results for the estimated effect of exposure. In all scenarios we also consider the coverage probability of the confidence intervals based on the unadjusted estimates of the standard errors, which do not account for the additional variation caused by including the estimated first stage residuals as covariates in the second stage. The results of the two continuous exposure Scenarios 1 and 2 are shown in Table 1. For Scenario 1 both two‐stage methods can be seen to be unbiased and near nominal coverage probabilities. The naive method has a substantial bias for all sample sizes and very small coverage probability that tends to 0 as the sample size increases. In Scenario 2 with exposure‐dependent censoring the 2SLS method is now biased. In Scenario 3, where the linearity assumption for the confounder is violated, 2SRI has a substantial bias, but the coverage probabilities are still close to the nominal level.

**Table 1:** Results of 50,000 simulations for scenarios 1–3 (continuous exposure) of benchmark (all confounders observed), two‐stage residual inclusion (2SRI), two‐stage least‐squares (2SLS), and naive (confounders ignored) analysis for varying sample sizes *n*. RMSE, root mean‐squared error; SD, standard deviation; ESE, estimated standard error; ESE${}^{*}$, estimated unadjusted standard error of; CP, coverage probability of 95% confidence interval; CP${}^{*}$, coverage probability of unadjusted 95% confidence interval

Scenario	*n*	Method	RMSE	Bias	SD	ESE	ESE${}^{*}$	CP	CP${}^{*}$	Power (%)
1	400	Benchmark	1.031	0.005	1.031	1.029	1.029	0.949	0.949	7.1
		2SRI	1.124	−0.030	1.123	1.106	1.114	0.948	0.948	7.4
		2SLS	1.118	−0.015	1.118	1.122	1.121	0.952	0.951	6.6
		Naive	1.275	1.177	0.489	0.485	0.485	0.310	0.310	93.5
	800	Benchmark	0.713	0.004	0.713	0.716	0.716	0.951	0.951	10.3
		2SRI	0.775	−0.024	0.774	0.772	0.772	0.950	0.949	9.9
		2SLS	0.767	−0.006	0.767	0.776	0.776	0.953	0.953	9.4
		Naive	1.218	1.170	0.337	0.339	0.339	0.061	0.061	99.9
2	400	Benchmark	1.083	0.019	1.083	1.086	1.086	0.950	0.950	6.9
		2SRI	1.192	0.008	1.192	1.201	1.184	0.955	0.949	6.8
		2SLS	1.194	−0.137	1.186	1.184	1.182	0.949	0.949	5.2
		Naive	1.287	1.173	0.530	0.530	0.530	0.390	0.390	89.2
	800	Benchmark	0.753	0.006	0.753	0.757	0.757	0.951	0.951	9.8
		2SRI	0.823	−0.003	0.823	0.829	0.819	0.953	0.949	9.2
		2SLS	0.826	−0.136	0.815	0.818	0.818	0.949	0.949	6.6
		Naive	1.225	1.168	0.369	0.370	0.370	0.109	0.109	99.5
3	400	Benchmark	1.054	0.008	1.054	1.054	1.054	0.951	0.951	6.9
		2SRI	1.117	0.103	1.113	1.129	1.127	0.953	0.953	7.3
		2SLS	1.127	0.010	1.127	1.128	1.128	0.951	0.951	6.5
		Naive	1.047	0.007	1.047	1.048	1.048	0.951	0.951	6.9
	800	Benchmark	0.734	0.001	0.734	0.733	0.733	0.951	0.951	10.3
		2SRI	0.785	0.044	0.784	0.787	0.786	0.950	0.950	10.2
		2SLS	0.789	−0.001	0.789	0.787	0.787	0.949	0.949	9.4
		Naive	0.731	−0.001	0.731	0.731	0.731	0.951	0.951	10.2

The results of the binary exposure scenarios are shown in Table 2. In Scenario 4 with a logistic regression model in the first stage the 2SLS method is again substantially biased, while 2SRI method is practically unbiased. Although, both methods have a substantially larger root mean‐squared error than the benchmark method and the massively biased naive method. The results for Scenario 4 also show clearly that the unadjusted estimator underestimates standard errors resulting in coverage probabilities below the nominal level. In Scenario 5 with a probit model in the first stage the 2SRI is unbiased even though the first stage model is misspecified, while 2SLS has a small bias. In Scenario 6, which is the same as Scenario 5, but with exposure‐dependent censoring 2SRI remains unbiased, while the bias of 2SLS increases. There is a notable difference in the coverage probabilities of the adjusted and unadjusted confidence intervals for the exposure effects for the 2SRI method. In the binary scenarios both IV methods do substantially increase the variance of the estimates leading to a large loss of power compared to the benchmark method. This is a general feature of the two‐stage IV methods and not specific to our method.

**Table 2:** Results of 50,000 simulations for scenarios 4–6 (binary exposure) of benchmark (all confounders observed), two‐stage residual inclusion (2SRI), two‐stage least‐squares (2SLS), and naive (confounders ignored) analysis for varying sample sizes *n*. RMSE, root mean‐squared error; SD, standard deviation; ESE, estimated standard error; ESE${}^{*}$, estimated unadjusted standard error of; CP, coverage probability of 95% confidence interval; CP${}^{*}$, coverage probability of unadjusted 95% confidence interval

Scenario	*n*	Method	RMSE	Bias	SD	ESE	ESE${}^{*}$	CP	CP${}^{*}$	Power (%)
4	400	Benchmark	0.089	−0.000	0.089	0.088	0.088	0.951	0.951	99.1
		2SRI	0.236	−0.004	0.236	0.236	0.232	0.955	0.948	43.0
		2SLS	0.239	0.068	0.229	0.238	0.238	0.952	0.952	51.8
		Naive	0.088	−0.007	0.088	0.087	0.087	0.950	0.950	99.0
	800	Benchmark	0.062	−0.001	0.062	0.062	0.062	0.950	0.950	100.0
		2SRI	0.162	−0.001	0.162	0.161	0.160	0.952	0.949	70.8
		2SLS	0.173	0.067	0.160	0.166	0.166	0.943	0.943	82.1
		Naive	0.062	−0.007	0.061	0.061	0.061	0.949	0.949	100.0
5	400	Benchmark	2.005	0.028	2.005	1.994	1.994	0.949	0.949	24.8
		2SRI	4.622	0.001	4.622	4.583	4.413	0.955	0.939	8.5
		2SLS	4.766	0.117	4.765	4.775	4.767	0.955	0.954	8.2
		Naive	2.668	2.211	1.493	1.480	1.480	0.674	0.674	88.8
	800	Benchmark	1.395	−0.013	1.394	1.391	1.391	0.949	0.949	43.4
		2SRI	3.149	0.005	3.149	3.142	3.084	0.953	0.945	13.0
		2SLS	3.262	0.132	3.260	3.284	3.283	0.953	0.952	12.7
		Naive	2.416	2.181	1.040	1.037	1.037	0.440	0.440	99.4
6	400	Benchmark	2.165	−0.055	2.165	2.168	2.168	0.952	0.952	21.1
		2SRI	4.809	−0.031	4.809	4.769	4.192	0.950	0.899	8.1
		2SLS	4.681	−0.289	4.672	4.677	4.672	0.952	0.952	7.2
		Naive	2.607	2.095	1.552	1.550	1.550	0.709	0.709	82.8
	800	Benchmark	1.518	−0.021	1.518	1.518	1.518	0.951	0.951	37.8
		2SRI	3.272	−0.036	3.272	3.264	3.024	0.950	0.925	11.9
		2SLS	3.231	−0.266	3.220	3.238	3.237	0.951	0.951	10.4
		Naive	2.379	2.116	1.087	1.088	1.088	0.495	0.495	98.2

For each of the seven scenarios we also used the 2SRI method to estimate the conditional survival function S(t|R=r,G=g,L=l) (with covariate values fixed at their mean values in the continuous scenarios). From the estimate $\hat{S}(t|r,g,l)$ the median is estimated as $\hat{m}=\text{inf}\{t:\hat{S}(t|r,g,l)>0.5\}$. The confidence interval for the median is obtained by inverting the pointwise confidence interval for $\hat{S}(\hat{m}|r,g,l)$. Simultaneous confidence bands for {S(t|R=r,G=g,L=l):t≤τ} were obtained using the bootstrap method from Theorem 2.3, where τ is chosen in each scenario such that on average approx. 10% of the subjects were still at risk at time τ.

The results are shown in Table 3. In all scenarios, the median estimate has a very small bias and the coverage probabilities are close to the nominal level. Only in Scenario 6, where the linearity assumption for the confounder (Assumption (A5c)) is violated, is the coverage probability of the simultaneous confidence band markedly below the nominal level.

**Table 3:** Mean of estimated median and 95% confidence intervals of the conditional survival function S(t|R,G,L) for scenarios 1–7 and sample sizes n=400 and 800 in 10,000 simulations. Coverage probabilities of 95% confidence intervals for the true median *m* (${\mathrm{CP}}_{m}$) and simultaneous confidence bands (${\mathrm{CP}}_{S}$) for the survival curve on [0,τ]. Simultaneous confidence bands are estimated from 1000 bootstrap replications

Scenario	τ	*m*	*n*	Median (95%CI)	${\mathrm{CP}}_{m}$ (%)	${\mathrm{CP}}_{S}$ (%)	
1	0.19	0.069	400	0.070 (0.060, 0.081)	94.7	94.6	
			800	0.069 (0.062, 0.077)	95.3	95.2	
2	0.17	0.069	400	0.070 (0.059, 0.081)	95.3	94.6	
			800	0.069 (0.062, 0.077)	95.2	94.8	
3	0.18	0.070	400	0.070 (0.060, 0.081)	94.9	94.1	
			800	0.070 (0.062, 0.077)	95.2	95.0	
4	2.50	0.770	400	0.775 (0.658, 0.905)	94.5	94.4	
			800	0.772 (0.689, 0.862)	95.0	95.0	
5	0.15	0.050	400	0.050 (0.041, 0.060)	94.8	94.5	
			800	0.050 (0.043, 0.057)	94.9	94.9	
6	0.12	0.050	400	0.048 (0.040, 0.058)	93.4	92.5	
			800	0.049 (0.043, 0.056)	93.1	92.7	
7	0.17	0.050	400	0.050 (0.041, 0.060)	95.0	94.8	
			800	0.050 (0.044, 0.058)	94.8	94.9	

**Figure 2:**
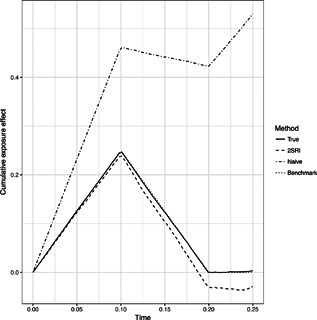
Results of Scenario 7. Mean of ${\hat{B}}_{R}(t)$ for t∈[0,0.25] of 10,000 simulations with sample size n=1000.

For Scenario 7 with the time‐dependent exposure effect the mean of the cumulative effect $B_{R}(t)=\int_{0}^{t}\beta_{R}(s)\mathrm{ds}$ is shown in Figure 2. Here, the naive method is substantially biased and fails to capture the true time‐dependency of the exposure. The 2SRI method is slightly biased for larger times as the number‐at‐risk becomes small. Web Figure 1 in Web Appendix A shows the means of the estimated survival functions S(t|R=0,G=0) and S(t|R=1,G=0), respectively, for a sample size of 1000. The 95% simultaneous confidence bands obtained from 1000 resampled processes have coverage probabilities 95.0% and 95.9%, respectively.

## 4 Application

We consider data from a social experiment conducted by Illinois Department of Employment Security between mid‐1984 and mid‐1985 to test the effect of cash bonuses in reducing the duration of insured unemployment (W.E. Upjohn Institute, 1987; Woodbury and Spiegelman, 1987). A total of 12,101 new claimants for unemployment insurance were randomized into 3 groups, 3952 to the control group (no cash bonus offered), 3963 to the employer bonus group (cash bonus offered to the next employer), and 4186 to the claimant bonus group (cash bonus offered to the claimant). The cash bonus of $500 was only paid if the claimants found a new job within 11 weeks of claiming unemployment insurance. Thus, it is plausible to assume that the effect of offering the bonus on the duration of unemployment is time‐dependent.

We will only analyze the data from the claimant bonus experiment consisting of the control group and the claimant bonus group. Subjects randomized to the control group were not informed about the experiment and not asked whether they wanted to participate. In the claimant bonus group 659 (15.7%) refused to participate for unknown reasons, which suggests that there is unobserved confounding.

This dataset has been previously analyzed using a two‐stage IV method based on a mixed proportional hazards model using the original randomization as the instrument (Bijwaard and Ridder, 2005). We analyze the dataset using the 2SLS and 2SRI methods, both with the cash bonus offer effect modeled as time‐dependent and time‐independent, and the naive method without any adjustment. The 2SRI is implemented based on the model in equation (4), which does not include the main effect of the first stage residual, since including the main effect made the design matrix singular for all event times. Following Bijwaard and Ridder (2005) we include age, the logarithm of pre‐unemployment earnings, gender, ethnicity, and the logarithm of the weekly amount of unemployment insurance benefits plus dependence allowance as additional covariates in our first and second stage models.

We use a formal goodness‐of‐fit test of the additive hazard model which has been proposed by Gandy and Jensen (2005). Their test statistic can be interpreted as a scaled sum of martingale residuals. The goodness‐of‐fit test indicates that the additive hazard model fits the data well for the female subgroup (p=0.14), but neither the male subgroup (p=0.006) nor the entire group ($p=1.7\times 10^{-5}$). We therefore restrict our analysis to the 3619 female participants in the claimant bonus experiment.

The estimated cumulative effects are shown in Web Figure 2 in Web Appendix A. The non‐parametric two‐stage estimates are slightly larger than the non‐parametric naive estimate. The 2SRI method in the McKeague–Sasieni model and the 2SLS method in the Lin–Ying model give practically identical results for the effect of the cash bonus offer with the estimated effect $2.84\times 10^{-3}$ with standard error $1.19\times 10^{-3}$ about 77.5% larger than the naive estimate $1.60\times 10^{-3}$ with standard error $1.02\times 10^{-3}$. All estimates are positive, that is, offering the cash bonus increases the hazard of re‐employment therefore shortening the duration of uninsurance benefit claims, as expected.

The estimated effect for the 2SRI method is statistically significant (p=0.008), but not for the naive method (p=0.059).

## 5 Discussion

We have provided asymptotic results for the two‐stage residual inclusion method in an semi‐parametric additive hazard model for binary and continuous exposure. These results include as a special case the general model where all effects are time‐dependent. The advantage of the semi‐parametric model in connection with 2SRI method is that the effect of the included residual may be time‐dependent, while the effect of other covariates can modeled as constant over time.

Our simulations have shown that the 2SRI method avoids the bias of 2SLS when censoring depends on the exposure and when the first stage is a non‐linear model. Although the asymptotic results assume a logistic regression model in the first stage, an extension to other generalized linear models would be straightforward. The coverage probabilities of the confidence intervals are near the nominal level even for relatively small sample sizes and the method is seen to be robust when the data is generated from a probit model in the first stage instead of the assumed logistic model. The naive method, which ignores any confounding, had in some cases a very large bias and coverage probabilities far below the nominal level.

A potential application of the 2SRI method is when drop‐out is suspected to depend on the level of exposure and/or the instrument, as this would be adjusted for.

It can be seen that the coverage probabilties of the confidence intervals based on the unadjusted standard errors can be substantially below the nominal level. This is despite the difference between the adjusted and unadjusted standard errors seemingly becoming smaller as the sample size increases.

## 6 Supplementary Materials

Figures referenced in Sections 3 and 4 and the R code (R Core Team, 2017) for fitting the two‐stage methods to the data set in Section 4 are available with this article at the *Biometrics* website on Wiley Online Library.

## Supporting information

Supplementary Data S1.

Supplementary Data Code S1.

## Appendix 1

### First Stage iid Decompositions

We state two well known asymptotic results for the maxmium‐likelihood estimators for the logistic and linear regression models, that we need for our proof of Theorem 2.1. We have

(A.1) $$\sqrt{n}(\hat{\gamma }-\gamma )=n^{-1/2}\sum_{i=1}^{n}\varepsilon_{i}^{\left(\gamma \right)}+o_{p}(1),$$

where $\varepsilon_{i}^{\left(\gamma \right)}$ (i=1,…,n) are independent and identically distributed mean‐zero random (p+q+2)‐vectors.

1. Logisitic regression: $\varepsilon_{i}^{\left(\gamma \right)}=V_{1}^{-1}{\left(1,G_{i},L_{i}^{\prime }\right)}^{\prime }\Delta_{i}$, where $V_{1}=-E\{p(1-p){\left(1,G,L^{\prime }\right)}^{\prime }(1,G,L^{\prime })\}$.
2. Linear regression: $\varepsilon_{i}^{\left(\gamma \right)}=V_{1}^{-1}{\left(1,G_{i},L_{i}^{\prime }\right)}^{\prime }\Delta_{i}$, where $V_{1}=E\{{\left(1,G,L^{\prime }\right)}^{\prime }(1,G,L^{\prime })\}$.

### Regularity Assumptions

A number of regularity assumptions are needed for proving our asymptotic results:

B1 There exist positive definite (p+1)×(p+1) matrices Ω and Σ such that $n^{-1}\int_{0}^{\tau }Z{\left(t\right)}^{\prime }H(t)Z(t)\mathrm{dt}\overset{p}{⟶}\Omega$ and $n^{-1}\int_{0}^{\tau }Z{\left(t\right)}^{\prime }H(t)\mathrm{diag}\{\mathrm{dN}(t)\}H{\left(t\right)}^{\prime }Z(t)\overset{p}{⟶}\Sigma$, where $H=I-X{\left(X^{\prime }X\right)}^{-1}X^{\prime }$.
B2 For k=1,2 exist positive definite matrices $\Gamma_{1k}$ such that
  $$n^{-1}\int_{0}^{\tau }Z{\left(t\right)}^{\prime }H(t)\mathrm{diag}\left\{u_{k}(t)\right\}X_{1}{\mathrm{dA}}_{\left(q+1+k\right)}(t)\overset{p}{⟶}\Gamma_{1k},$$
  where $X_{1}$ is the n×r design matrix of the first stage regression, and $u_{1}(t)$ and $u_{2}(t)$ are vectors defined by $u_{1i}(t)=p_{i}(1-p_{1})Y_{i}(t)$ and $u_{2i}(t)=u_{1i}(t)G_{i}$, respectively. Let $\Gamma_{1}=\Gamma_{11}+\Gamma_{12}$.
B3 The covariates R,G, and *L* have bounded support.

In order to prove uniform consistency of *A* and convergence of $\sqrt{n}(\hat{A}-A)$ to a mean‐zero Gaussian process we need the following additional assumptions:

B4 There exists a positive definite (q+3)×(q+3) matrix function ξ(t) such that
  $$\begin{array}{ccc} & & n\underset{t}{\text{sup}}∥\int_{0}^{t}{\left\{X{\left(s\right)}^{\prime }X(s)\right\}}^{-1}X{\left(s\right)}^{\prime }\mathrm{diag}\left\{\mathrm{dN}(s)\right\}X(s)\\ & & \;\times {\left\{X{\left(s\right)}^{\prime }X(s)\right\}}^{-1}-\xi (t)∥\overset{p}{⟶}0. \end{array}$$
B5 There exists positive definite (p+1)×(q+3) matrices Ψ(t) such that
  $$\underset{t}{\text{sup}}∥\int_{0}^{t}{\left\{X{\left(s\right)}^{\prime }X(s)\right\}}^{-1}X{\left(s\right)}^{\prime }Z(s)\mathrm{ds}-\Psi (t)∥\overset{p}{⟶}0,$$
  where $∥A∥=\underset{i}{\text{max}}\sum_{j}|a_{\mathrm{ij}}|$ for a matrix $A=(a_{\mathrm{ij}})$.
B6 For k=1,2 and t∈[0,τ] exist positive definite matrices $\Gamma_{2k}(t)$ such that
  $$\begin{array}{ccc} & & \underset{t}{\text{sup}}∥n^{-1}\int_{0}^{t}{\left\{X{\left(s\right)}^{\prime }X(s)\right\}}^{-1}X{\left(s\right)}^{\prime }\mathrm{diag}\left\{u_{k}(s)\right\}\\ & & \;\times X_{1}{\mathrm{dA}}_{\left(q+1+k\right)}(s)-\Gamma_{2k}(t)∥\overset{p}{⟶}0. \end{array}$$
  Let $\Gamma_{2}(t)=\Gamma_{21}(t)+\Gamma_{22}(t)$ for t∈[0,τ].

Furthermore, we also assume the regularity conditions required for asymptotic normality of the maximum‐likelihood estimator $\hat{\gamma }$ in the logistic regression model. Specifically, we assume that

(A.2) $$\sqrt{n}(\hat{\gamma }-\gamma )=n^{-1/2}\sum_{i=1}^{n}\varepsilon_{i}^{\left(\gamma \right)}+o_{p}(1),$$

where $\varepsilon_{i}^{\left(\gamma \right)}$ are iid random variables defined in the Appendix with covariance matrix $K=E\{\varepsilon_{i}^{(\gamma )\prime }\varepsilon_{i}^{\left(\gamma \right)}\}$.

### Proofs

Lemma 1 Under Assumptions (B1), (B3), and (B4)

$$n^{-1/2}\int_{0}^{\tau }Z^{\prime }\hat{H}(\hat{X}-X)\mathrm{dA}=-\Gamma_{1}n^{-1/2}\sum_{j=1}^{n}\varepsilon_{j}^{\left(\gamma \right)}+o_{p}(1),$$

and

$$n^{-1/2}\int_{0}^{t}{\left({\hat{X}}^{\prime }\hat{X}\right)}^{-1}{\hat{X}}^{\prime }(\hat{X}-X)\mathrm{dA}=-\Gamma_{2}(t)n^{-1/2}\sum_{j=1}^{n}\varepsilon_{j}^{\left(\gamma \right)}+o_{p}(1).$$

We only prove the first equation, the proof of the second is almost identical. We have $\hat{X}(t)=X(t)+\{0_{n\times (q+1)},v_{1}(t),v_{2}(t)\}$, where $v_{1i}(t)=Y_{i}(t)({\hat{\Delta }}_{i}-\Delta_{i})=-Y_{i}(t)({\hat{p}}_{i}-p_{i})$ and $v_{2i}(t)=v_{1i}(t)G_{i}$. The delta method implies $\sqrt{n}({\hat{p}}_{i}-p_{i})=p_{i}(1-p_{i})(1,G_{i},L_{i}^{\prime })\sqrt{n}(\hat{\gamma }-\gamma )+o_{p}(1)$. Therefore,

$$\begin{array}{c} n^{-1/2}\int_{0}^{\tau }Z^{\prime }\hat{H}(\hat{X}-X)\mathrm{dA}\\ =n^{-1/2}\int_{0}^{\tau }Z^{\prime }\hat{H}\{v_{1}(t){\mathrm{dA}}_{\left(q+2\right)}(t)+v_{2}(t){\mathrm{dA}}_{\left(q+3\right)}(t)\}\\ =-n^{-1}\int_{0}^{\tau }Z^{\prime }\hat{H}\left[\mathrm{diag}\left\{u_{1}(t)\right\}X_{1}{\mathrm{dA}}_{\left(q+2\right)}(t)\right.\\ \left.\;+\mathrm{diag}\left\{u_{2}(t)\right\}X_{1}{\mathrm{dA}}_{\left(q+3\right)}(t)\right]\sqrt{n}(\hat{\gamma }-\gamma )+o_{p}(1) \end{array}$$

Since the covariates are bounded and $\hat{\gamma }=\gamma +o_{p}(1)$ we have $\underset{t}{\text{sup}}∥Z^{\prime }\hat{H}-Z^{\prime }H∥=o_{p}(1)$. The conclusion then follows by Assumption (B3) and equation (A.2).

[Proof of Theorem 1] Let $M={\left(M_{1},\ldots ,M_{n}\right)}^{\prime }$ be the vector of counting process martingales, where

$$M_{i}(t)=N_{i}(t)-\int_{0}^{t}Y_{i}(s)h(s)\mathrm{ds}.$$

We have

$$\begin{array}{ccc} \begin{array}{cc} \int_{0}^{\tau }Z^{\prime }\hat{H}\mathrm{dM} & =\int_{0}^{\tau }Z^{\prime }\hat{H}\mathrm{dN}-\int_{0}^{\tau }Z^{\prime }\hat{H}\mathrm{XdA}-\int_{0}^{\tau }Z^{\prime }\hat{H}\mathrm{Zdt}\beta \\ & =\int_{0}^{\tau }Z^{\prime }\hat{H}\mathrm{dN}+\int_{0}^{\tau }Z^{\prime }\hat{H}(\hat{X}-X)\mathrm{dA}-\int_{0}^{\tau }Z^{\prime }\hat{H}\mathrm{Zdt}\beta \end{array} & & \end{array}$$

since $\hat{H}\hat{X}=0$. Thus,

$$\begin{array}{ccc} \sqrt{n}\beta & = & {\left(n^{-1}\int_{0}^{\tau }Z^{\prime }\hat{H}\mathrm{Zdt}\right)}^{-1}n^{-1/2}\left\{\int_{0}^{\tau }Z^{\prime }\hat{H}\mathrm{dN}\right.\\ & & \left.+\int_{0}^{\tau }Z^{\prime }\hat{H}(\hat{X}-X)\mathrm{dA}-\int_{0}^{\tau }Z^{\prime }\hat{H}\mathrm{dM}\right\}, \end{array}$$

and with the definition of $\hat{\beta }$ in equation (5) we have

(A.3) $$\begin{array}{ccc} \sqrt{n}(\hat{\beta }-\beta ) & = & {\left(n^{-1}\int_{0}^{\tau }Z^{\prime }\hat{H}\mathrm{Zdt}\right)}^{-1}n^{-1/2}\int_{0}^{\tau }Z^{\prime }\hat{H}\mathrm{dM}\\ & & -{\left(n^{-1}\int_{0}^{\tau }Z^{\prime }\hat{H}\mathrm{Zdt}\right)}^{-1}n^{-1/2}\int_{0}^{\tau }Z^{\prime }\hat{H}(\hat{X}-X)\mathrm{dA} \end{array}$$

Since $\underset{t}{\text{sup}}∥\hat{X}(t)-X(t)∥=o_{p}(1)$ we have $\underset{t}{\text{sup}}∥n^{-1}Z^{\prime }\hat{H}Z-n^{-1}Z^{\prime }\mathrm{HZ}∥=o_{p}(1)$. By Assumption (B1) and Lemma 1 the second term on the right hand side becomes

(A.4) $$\Omega^{-1}\Gamma_{1}n^{-1/2}\sum_{j=1}^{n}\varepsilon_{j}^{\left(\gamma \right)}+o_{p}(1),$$

which is a sum of mean‐zero iid terms and asymptotic normality follows from the central limit theorem. Asymptotic normality of the first term on the right hand side of equation (A.3) follows from the martingale central limit theorem (Andersen et al., 1993). The asymptotic variance of $\sqrt{n}(\hat{\beta }-\beta )$ follows, since the two terms on the right hand side of equation (A.3) are asymptotically independent. Thus, $\Sigma_{\beta }=\Omega^{-1}\{\Sigma +\Gamma_{1}K\Gamma_{1}^{\prime }\}\Omega^{-1}$. For later reference, we note that $\sqrt{n}(\hat{\beta }-\beta )$ admits the following iid decomposition $\sqrt{n}(\hat{\beta }-\beta )=n^{-1/2}\sum_{i}\varepsilon_{i}^{\left(\beta \right)}+o_{p}(1)$, where

(A.5) $$\varepsilon_{i}^{\left(\beta \right)}=\Omega^{-1}\int_{0}^{\tau }\left\{Z_{\cdot i}^{\prime }(t)-\psi {\left(t\right)}^{\prime }X_{\cdot i}^{\prime }(t)\right\}dM_{i}(t)+\Omega^{-1}\Gamma_{1}\varepsilon_{i}^{\left(\gamma \right)},$$

where $\psi {\left(t\right)}^{\prime }=Z{\left(t\right)}^{\prime }X(t)\{X{\left(t\right)}^{\prime }X(t){\;\}}^{-1}$. Now let $\hat{Q}={\left({\hat{X}}^{\prime }\hat{X}\right)}^{-1}{\hat{X}}^{\prime }$, $Q={\left(X^{\prime }X\right)}^{-1}X^{\prime }$. For showing asymptotic normality of $\hat{A}$ we start by noting that

$$\begin{array}{ccc} \begin{array}{cc} \sqrt{n}\{\hat{A}(t)-A(t)\}= & \sqrt{n}\int_{0}^{t}\hat{Q}\mathrm{dN}-\int_{0}^{t}\hat{Q}\mathrm{Zds}\sqrt{n}(\hat{\beta }-\beta )\\ & -\sqrt{n}\int_{0}^{t}\hat{Q}\hat{X}\mathrm{dA}-\sqrt{n}\int_{0}^{t}\hat{Q}Z\beta \mathrm{ds}+o_{p}(1)\\ = & \sqrt{n}\int_{0}^{t}\hat{Q}\mathrm{dM}-\sqrt{n}\int_{0}^{t}\hat{Q}(\hat{X}-X)\mathrm{dA}\\ & -\int_{0}^{t}\hat{Q}\mathrm{Zds}\sqrt{n}(\hat{\beta }-\beta )+o_{p}(1) \end{array} & & \end{array}$$

The second term on the right hand side is asymptotically equivalent to $\Gamma_{2}(t)n^{-1/2}\sum_{j=1}^{n}\varepsilon_{j}^{\left(\gamma \right)}$, by Lemma 1, and the last term is asymptotically equivalent to $-\Psi {\left(t\right)}^{\prime }n^{-1/2}\sum_{i}\varepsilon_{i}^{\left(\beta \right)}$ by Assumption (B2) and equation (A.4). Thus,

(A.6) $$\begin{array}{ccc} \sqrt{n}\{\hat{A}(t)-A(t)\} & = & \sqrt{n}\int_{0}^{t}\hat{Q}\mathrm{dM}-\Psi {\left(t\right)}^{\prime }n^{-1/2}\sum_{i=1}^{n}\varepsilon_{i}^{\left(\beta \right)}\\ & & +\Gamma_{2}(t)n^{-1/2}\sum_{j=1}^{n}\varepsilon_{j}^{\left(\gamma \right)}+o_{p}(1). \end{array}$$

The martingale central limit theorem and Assumption (B3) imply convergence of the first term on the right hand side to a mean‐zero Gaussian process with covariation function (s,t)↦ξ(s∧t). The second term converges to a mean‐zero Gaussian process $\Psi {\left(\cdot \right)}^{\prime }m_{\beta }$ where $m_{\beta }$ is a mean‐zero normal random vector with covariance matrix $\Omega^{-1}\Sigma \Omega^{-1}$. The last term on the right hand side converges to a mean‐zero Gaussian process $\{\Gamma_{2}(\cdot )-\Psi {\left(\cdot \right)}^{\prime }\Omega^{-1}\Gamma_{1}\}m_{r}$ where $m_{r}$ is a mean‐zero normal random vector with covariance matrix *K*. All three processes are asymptotically independent, since each $\varepsilon_{j}^{\left(\gamma \right)}$ is time‐independent and the covariation between the two martingale processes is 0, by a similar argument as that in Appendix 1 of McKeague and Sasieni (1994). Thus, $\Sigma_{A}(s,t)=\xi (s\wedge t)+\Psi (s)\Sigma_{\beta }\Psi {\left(t\right)}^{\prime }+\Gamma_{2}(s)K\Gamma_{2}{\left(t\right)}^{\prime }$. In order to prove uniform consistency of $\hat{A}$ on [0,τ] we divide equation (A.6) by $\sqrt{n}$ and see that all terms converge to 0 uniformly in probability, the first two terms by Lenglart's inequality (Andersen et al., 1993) and the last term because of the law of large numbers. We have again an iid decomposition $\sqrt{n}\{\hat{A}(t)-A(t)\}=n^{-1/2}\sum_{i}\varepsilon_{i}^{\left(A\right)}(t)+o_{p}(1)$, where

(A.7) $$\varepsilon_{i}^{\left(A\right)}(t)=\int_{0}^{t}V^{-1}X_{\cdot i}^{\prime }dM_{i}-\Psi {\left(t\right)}^{\prime }\varepsilon_{i}^{\left(\beta \right)}+\Gamma_{2}(t)\varepsilon_{i}^{\left(\gamma \right)}.$$

## References

[biom12952-bib-0001] Aalen, O. O. (1989). A linear regression model for the analysis of life times. Statistics in Medicine 8, 907–925. 2678347 10.1002/sim.4780080803

[biom12952-bib-0002] Amado, R. G. , Wolf, M. , Peeters, M. , VanCutsem, E. , Siena, S. , Freeman, D. J. , et al. (2008). Wild‐type KRAS is required for panitumumab efficacy in patients with metastatic colorectal cancer. Journal of Clinical Oncology 26, 1626–1634. 18316791 10.1200/JCO.2007.14.7116

[biom12952-bib-0003] Andersen, P. K. , Gill, R. D. , and Keiding, N. (1993). Statistical Models Based on Counting Processes. Springer Series in Statistics. Springer‐Verlag, New York.

[biom12952-bib-0004] Angrist, J. D. , Imbens, G. W. , and Rubin, D. B. (1996). Identification of causal effects using instrumental variables. Journal of the American Statistical Association 91, 444–455.

[biom12952-bib-0005] Baker, S. G. (1998). Analysis of survival data from a randomized trial with all‐or‐none compliance: Estimating the cost‐effectiveness of a cancer screening program. Journal of the American Statistical Association 93, 929–934.

[biom12952-bib-0006] Bijwaard, G. E. and Ridder, G. (2005). Correcting for selective compliance in a re‐employment bonus experiment. Journal of Econometrics 125, 77–111.

[biom12952-bib-0007] Chan, K. C. G. (2016). Reader reaction: Instrumental variable additive hazards models with exposuredependent censoring. Biometrics 72, 1003–1005. 26754156 10.1111/biom.12471 PMC4940314

[biom12952-bib-0008] Choi, B. Y. , Fine, J. P. , and Brookhart, M. A. (2017). On two‐stage estimation of structural instrumental variable models. Biometrika 104, 881–899. 29430042 10.1093/biomet/asx056 PMC5793491

[biom12952-bib-0009] Gandy, A. and Jensen, U. (2005). On goodness‐of‐fit tests for Aalen's additive risk model. Scandinavian Journal of Statistics 32, 425–445.

[biom12952-bib-0010] Li, G. and Lu, X. (2015). A Bayesian approach for instrumental variable analysis with censored time‐to‐event outcome. Statistics in Medicine 34, 664–684. 25393617 10.1002/sim.6369 PMC4314427

[biom12952-bib-0011] Li, J. , Fine, J. , and Brookhart, A. (2015). Instrumental variable additive hazards models. Biometrics 71, 122–130. 25298257 10.1111/biom.12244

[biom12952-bib-0012] Lin, D. Y. and Ying, Z. (1994). Semiparametric analysis of the additive risk model. Biometrika 81, 61–71.

[biom12952-bib-0013] Martinussen, T. and Scheike, T. H. (2006). Dynamic Regression Models for Survival Data. Statistics for Biology and Health. New York: Springer‐Verlag.

[biom12952-bib-0014] Martinussen, T. , Vansteelandt, S. , Tchetgen, T.,J, E. , and Zucker, D. M. (2017). Instrumental variables estimation of exposure effects on a time‐to‐event endpoint using structural cumulative survival models. Biometrics 73, 1140–1149. 28493302 10.1111/biom.12699 PMC5681451

[biom12952-bib-0015] McKeague, I. W. and Sasieni, P. D. (1994). A partly parametric additive risk model. Biometrika 81, 501–514.

[biom12952-bib-0016] Nie, H. , Cheng, J. , and Small, D. S. (2011). Inference for the effect of treatment on survival probability in randomized trials with noncompliance and administrative censoring. Biometrics 67, 1397–1405. 21385167 10.1111/j.1541-0420.2011.01575.x

[biom12952-bib-0017] R Core Team (2017). R: A Language and Environment for Statistical Computing. R Foundation for Statistical Computing, Vienna, Austria.

[biom12952-bib-0018] Richardson, A. , Hudgens, M. G. , Fine, J. P. , and Brookhart, M. A. (2017). Nonparametric binary instrumental variable analysis of competing risks data. Biostatistics 18, 48–61. 27354709 10.1093/biostatistics/kxw023 PMC6497235

[biom12952-bib-0019] Tchetgen Tchetgen, E. J. , Walter, S. , Vansteelandt, S. , Martinussen, T. , and Glymour, M. (2015). Instrumental variable estimation in a survival context. Epidemiology (Cambridge, Mass.) 26, 402–410. 10.1097/EDE.0000000000000262 PMC4387894 25692223

[biom12952-bib-0020] Terza, J. V. , Basu, A. , and Rathouz, P. J. (2008). Two‐stage residual inclusion estimation: Addressing endogeneity in health econometric modeling. Journal of Health Economics 27, 531–543. 18192044 10.1016/j.jhealeco.2007.09.009 PMC2494557

[biom12952-bib-0021] W.E. Upjohn Institute (1987). The Illinois Unemployment Insurance Experiments public use data. https://upjohn.org/node/950. Accessed: 2017‐06–29.

[biom12952-bib-0022] Woodbury, S. A. and Spiegelman, R. G. (1987). Bonuses to workers and employers to reduce unemployment: Randomized trials in illinois. The American Economic Review 77, 513–530.

[biom12952-bib-0023] Zeng, D. , Chen, Q. , Chen, M.‐H. , Ibrahim, J. G. , and Groups, A. R. (2012). Estimating treatment effects with treatment switching via semicompeting risks models: An application to a colorectal cancer study. Biometrika 99, 167–184. 23049136 10.1093/biomet/asr062 PMC3412606

