# Supplementary material for: Instrumental variable estimation in semi‐parametric additive hazards models
Source: Biometrics. 2018 Aug 2;75(1):110–20. doi: 10.1111/biom.12952 (PMC7379316; doi:10.1111/biom.12952)
Supplement: Supplementary file 1 — Supplementary Data S1. [file BIOM-75-110-s001.pdf]

BIOMETRICS 000, 000–000 DOI: 000
000 0000

Web-based Supplementary Materials for Instrumental variable estimation in semi-parametric additive hazards models by Matthias Brueckner, Andrew Titman and Thomas Jaki

This paper has been submitted for consideration for publication in *Biometrics*

Web Appendix A

[Figure 1 about here.]

[Figure 2 about here.]

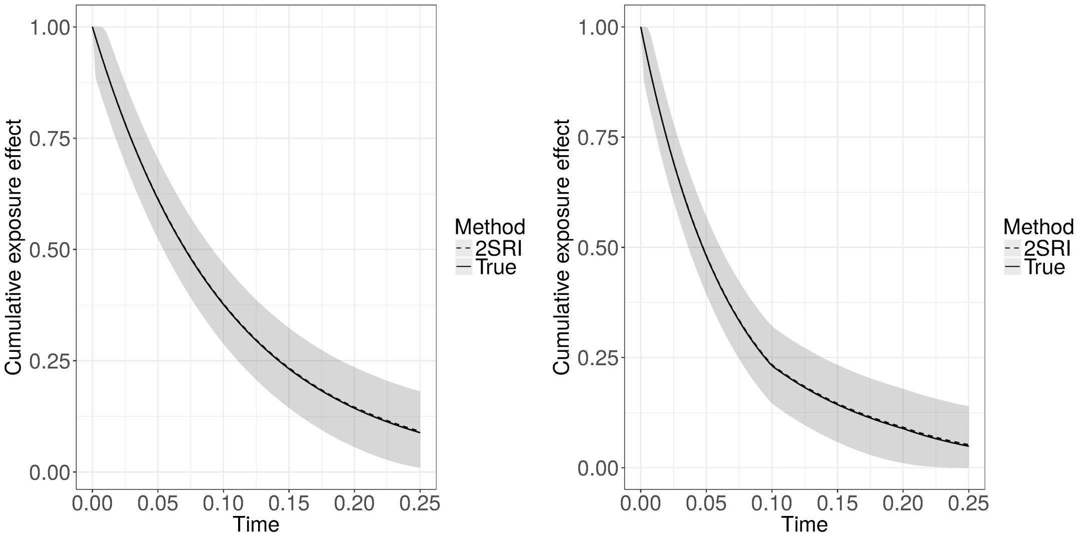

Figure 1. Results of Scenario 5. Mean of predicted survival functions and simultaneous confidence bands for $R = 0, G = 0$ (left) and $R = 1, G = 0$ (right) in 10000 simulations with sample size $n = 1000$ and 1000 bootstrap samples each. Coverage probabilities of confidence bands are 95.0% (left) and 95.9% (right), respectively.

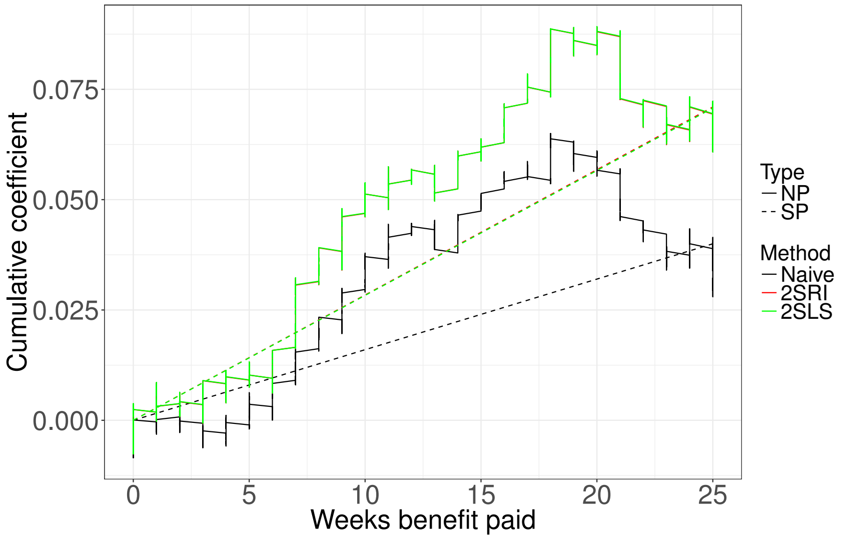

Figure 2. Cumulative effect of cash bonus offer on the unemployment duration hazard in the Illinois Unemployment Insurance Experiment.
